# Managing Small-Scale Commercial Fisheries for Adaptive Capacity: Insights from Dynamic Social-Ecological Drivers of Change in Monterey Bay

**DOI:** 10.1371/journal.pone.0118992

**Published:** 2015-03-19

**Authors:** Stacy E. Aguilera, Jennifer Cole, Elena M. Finkbeiner, Elodie Le Cornu, Natalie C. Ban, Mark H. Carr, Joshua E. Cinner, Larry B. Crowder, Stefan Gelcich, Christina C. Hicks, John N. Kittinger, Rebecca Martone, Daniel Malone, Carrie Pomeroy, Richard M. Starr, Sanah Seram, Rachel Zuercher, Kenneth Broad

**Affiliations:** 1 Leonard and Jayne Abess Center for Ecosystem Science and Policy, University of Miami, 1365 Memorial Drive, Ungar Building 230M, Coral Gables, FL 33124, United States of America; 2 Hopkins Marine Station, Stanford University, 120 Oceanview Blvd., Pacific Grove, CA 93950, United States of America; 3 Center for Ocean Solutions, Stanford University, Stanford Woods Institute for the Environment, 99 Pacific Street, Suite 555E, Monterey, CA 93940, United States of America; 4 School of Environmental Studies, University of Victoria, PO Box 1700 STN CSC, Victoria, BC V8W 2Y2, Canada; 5 Ecology and Evolutionary Biology, University of California Santa Cruz, Long Marine Lab, 100 Shaffer Road, Santa Cruz, CA 95060, United States of America; 6 Australian Research Council Centre of Excellence for Coral Reef Studies, James Cook University, Queensland 4811, Australia; 7 Center of Applied Ecology and Sustainability (CAPES) & Centro de Conservacion Marina, Departamento de Ecología, Facultad de Ciencias Biologicas, Pontificia Universidad Católica de Chile, Casilla 114-D, Chile; 8 Conservation International, Betty and Gordon Moore Center for Science and Oceans, 7192 Kalanianaole Hwy, Ste G-230, Honolulu, Hawaii 96825, United States of America; 9 University of California Sea Grant Extension Program and Institute of Marine Sciences, University of California Santa Cruz, Center for Ocean Health, 100 Shaffer Road, Santa Cruz, CA 95060, United States of America; 10 University of California Sea Grant Extension Program, Moss Landing Marine Laboratories, 8272 Moss Landing Road, Moss Landing, California 95039, United States of America; 11 McGill School of Environment, McGill University, 3534 University Street, Montreal, Quebec, H3A 2A7, Canada; University of Waterloo, CANADA

## Abstract

Globally, small-scale fisheries are influenced by dynamic climate, governance, and market drivers, which present social and ecological challenges and opportunities. It is difficult to manage fisheries adaptively for fluctuating drivers, except to allow participants to shift effort among multiple fisheries. Adapting to changing conditions allows small-scale fishery participants to survive economic and environmental disturbances and benefit from optimal conditions. This study explores the relative influence of large-scale drivers on shifts in effort and outcomes among three closely linked fisheries in Monterey Bay since the Magnuson-Stevens Fisheries Conservation and Management Act of 1976. In this region, Pacific sardine (*Sardinops sagax*), northern anchovy (*Engraulis mordax*), and market squid (*Loligo opalescens*) fisheries comprise a tightly linked system where shifting focus among fisheries is a key element to adaptive capacity and reduced social and ecological vulnerability. Using a cluster analysis of landings, we identify four modes from 1974 to 2012 that are dominated (i.e., a given species accounting for the plurality of landings) by squid, sardine, anchovy, or lack any dominance, and seven points of transition among these periods. This approach enables us to determine which drivers are associated with each mode and each transition. Overall, we show that market and climate drivers are predominantly attributed to dominance transitions. Model selection of external drivers indicates that governance phases, reflected as perceived abundance, dictate long-term outcomes. Our findings suggest that globally, small-scale fishery managers should consider enabling shifts in effort among fisheries and retaining existing flexibility, as adaptive capacity is a critical determinant for social and ecological resilience.

## Introduction

Among fishing communities, fisheries diversification and occupational multiplicity are critical strategies fishermen use to respond to environmental, regulatory, and economic variability and change, and contribute to the viability and welfare of a fishery and associated communities [1–3]. It is common for fishermen to engage in many different fisheries, using a “portfolio approach,” shifting focus among fisheries in response to various social and ecological drivers [4–7]. Historically, fishermen have shifted effort among fisheries because of (1) management strategies that limit or promote particular gear types [8], (2) the availability of more valued or abundant species [7], (3) climate variability [9], and (4) ease of adapting one’s vessel, gear, or location [10]. However, because of regulatory and economic factors, fisheries diversification is declining in the US [11–13]. Recent changes in fisheries policy in the US, such as the implementation of limited access privilege programs (LAPPs, limited entry, catch shares) have been shown in some cases to reduce fisheries diversity and produce inequitable outcomes [13–15]. Such programs, often marketed as the professionalization of fisheries, can increase the cost of entry, such as permits, and thereby reduce the number of different fisheries a single fisherman might be able to access. Moreover, traditional management plans rarely specifically address interactions among fisheries, further hindering adaptive capacity [16]. This presents problems for sustaining fisheries in the face of stochastic disturbances such as disease outbreaks, market fluctuations, or long-term shifts in climate [17].

The ability to participate in a diversity (or portfolio) of fisheries is especially vital for small-scale fisheries (SSF) [1,18]. Globally, SSFs can be defined in many ways, but generally these fisheries target multiple species, are carried out by individuals, families, or small firms rather than by larger corporate entities, and typically have a lower environmental impact and less financial investment than industrial fisheries [18–21]. While often overlooked in national policies and statistics, SSFs contribute more than 120 million jobs (direct and indirect) that support more than 500 million people globally [22]. Possible compounding factors such as environmental fluctuations and human system dynamics can make it difficult to predict future conditions that affect SSFs, especially given the long temporal scales and large areas in which many fisheries function [23]. In systems that encourage flexibility, fishery participants are better positioned to take advantage of opportunities such as resource abundance and strong markets, and avoid or buffer themselves against challenges [24,25]. Common fisheries management approaches such as catch shares and other rights-based approaches can, however, make it more difficult to adapt [13,14]. Additionally, failure to account for important social factors that influence adaptive capacity has made many SSFs more vulnerable to external drivers [26].

### Adaptability in small-scale fisheries

The Monterey Bay commercial fisheries for Pacific sardine (*Sardinops sagax*, Clupeidae), northern anchovy (*Engraulis mordax*, Engraulidae), and market squid (*Loligo opalescens*, Loliginidae) comprise an interdependent system known as the “wetfish fisheries,” in which fishermen and buyers shift effort among the three individual species-specific fisheries in response to several drivers (Table 1). ‘Wetfish’ refers to species that are canned fresh or ‘wet’ and are then cooked inside the cans. We study the history of Monterey Bay wetfish fisheries as a case study in order to (1) explore an approach for analyzing adaptability in SSFs from a social-ecological perspective in a data-rich location, and (2) summarize lessons from this case study for other SSFs.

**Table 1:** Key features of the commercial fisheries that comprise the interconnected Monterey Bay wetfish fisheries system.

	Fishery		
	Market Squid	Northern Anchovy	Pacific Sardine
**Primary management authority**	State	Federal	Federal
**FMP implementation**	2005	1978	2000
**Limited entry implementation**	1998	2000	2000
**Limited entry permit type**	Squid	CPS Finfish	CPS Finfish
Number of permits, 2013*	76	61	61
**Number of resident vessels in area**	~10	~10	~10
**Number of resident seafood buyers in area**	4	4	4
**Primary gear**	Round haul net	Round haul net	Round haul net
**Peak season**	Spring/Summer	Fall	Fall
**Preferred oceanographic regime**	Cooler	Cooler	Warmer
**Spawning habitat**	Nearshore	Nearshore	Offshore
**Primary market destination**	China	Domestic US	Japan/Australia
**Average ex-vessel price, 1974–2012 ($/lb)**	0.245	0.062	0.148

*Available permits does not indicate the number of vessels with landings as some permitted vessels may not participate in a given year. The number of market squid permits applies only to round haul (seine) vessels; light boat and brail vessel permits are issued separately.

The social and ecological complexities of fisheries viewed as common pool resource systems call for an interdisciplinary approach to addressing these aims [27,28]. To do so, we identify key climate, governance, and market drivers that are associated with focus shifting among target species and we contextualize the social and ecological conditions that existed when system flexibility was utilized. We also identify the role of drivers associated with long-term fishery trends. By exemplifying time periods when fishermen have relied on the flexibility to adapt to reduce social and ecological vulnerability, we not only reinforce literature on the importance of flexibility in fishery systems, but also present an analysis applicable to other SSFs.

We focus on Monterey Bay’s SSF because the flexibility to adapt appears to be central to the persistence of these fisheries in the past four decades. These fisheries are considered fully recovered from the 1950s industrial sardine fishery collapse, and since the passage of the Magnuson Stevens Fisheries Conservation and Management Act (MSA) of 1976, have sustained relative ecological and economic stability and multi-generational participation.

This study aims to answer three major questions applied to the Monterey Bay case study as examples that can be applied to other SSF cases:

1) What drivers are associated with fishery participants shifting focus among strongly interconnected small-scale fisheries?
2) What drivers best explain disproportionate representation (low evenness) among species in fishery landings?
3) What drivers are associated with the total and relative contribution of the different target species landings over time?

### The Monterey Bay wetfish fisheries system

The sardine, anchovy, and squid small-scale fisheries share several key characteristics that contribute to the flexibility and connectivity of the Monterey Bay social-ecological system. First, in most cases, the same fishermen, crew, vessels, buyers, and shoreside receivers and processors participate in all three fisheries. Fishermen use similar fishing gear, methods, and practices in the three fisheries, making it relatively easy to shift effort among target species. All of these fisheries occur within three miles of the coast, and are carried out as day fisheries (as opposed to multi-day or ‘trip’ fisheries). Moreover, the seasonal availability of these species varies, with anchovy and sardine most abundant in the fall and squid available primarily in the spring and summer. Lastly, most fishery participants hold both a federal coastal pelagic species (CPS) permit and a state market squid limited entry permit, affording them the flexibility to participate in—and shift effort among—these fisheries [29].

This flexibility allows for the persistence of one of the most significant fisheries in California. In 2012, landings of Pacific sardine, northern anchovy, and market squid accounted for 91.3 percent of landings and 48.3 percent of ex-vessel revenue at the three major Monterey Bay ports (Monterey, Moss Landing, and Santa Cruz), and 77 percent of the total statewide commercial fishery landings by volume and 30 percent of total state ex-vessel value [30]. In 2011, 80.4 percent of total California seafood exports were wetfish, and totaled 195 million US dollars [29]. In 2011, approximately 31 vessel owners (defined as an individual with commercial fishery landings data associated with a commercial fishing license number) participated in the Central Coast region CPS fishery, 14 of whom were identified by CDFW as primarily participating at the Monterey port [31].

Despite the high volume of landings and ex-vessel revenue generated by these fisheries compared to other Monterey Bay area fisheries, we consider the Monterey Bay wetfish fisheries to be small-scale because of the individual ownership, targeting of multiple species, and involvement of family members. Vessels range between 30 and 90 feet in length, and between 20 and 140 gross registered tons [29]. Vessels are operated by 3 to 7 crewmembers [32].

### Fishery management structure

The Monterey Bay small-scale fisheries operate under the auspices of a complex state and federal management regime that includes consideration of fishing effort, stock abundance, environmental change, and the role of these species as prey items. Pacific sardine, northern anchovy, and market squid all fall under the Federal Coastal Pelagic Species (CPS) Fisheries Management Plan (FMP) [33]. The market squid fishery is managed by the state, and regulations are consistent with the federal CPS FMP as squid is a “monitored” species under the federal plan [34]. The Market Squid Fishery Management Plan (MSFMP) limited entry program reduced the fleet to 77 transferable purse seine vessel permits in 2005 [35]. About 45 to 50 seiners also carry the CPS finfish permits [29]. As of 2013, the federal CPS limited entry fleet consisted of 61 permittees, 33 of which landed sardine and 11 of which landed anchovy in California [36] (see S1 Text for additional information on the structure of the fisheries and their management).

## Methods

### Study design

**Fig 1:**
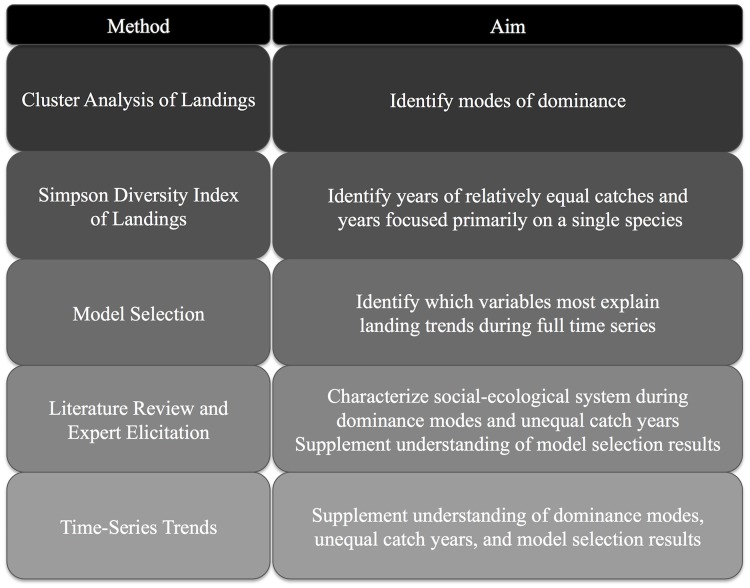
Aims of methods used.

This study addresses its aims using mixed methods, which are outlined in Fig. 1. In order to understand the level and role of adaptability in this case study, we first had to identify when focus shifted among fisheries, and then had to contextualize the status of the fisheries and conditions at the time of transitions between dominance modes. A cluster analysis and Simpson’s diversity index were used to identify modes of dominance. Model selection, literature review, use of expert knowledge, and time series plots of fishery information were used to contextualize the social and ecological conditions and identify drivers associated with landings trends. We use these methods to show quantitatively and qualitatively how the flexibility to adapt has led to the persistence of these fisheries through changing conditions.

**Fig 2:**
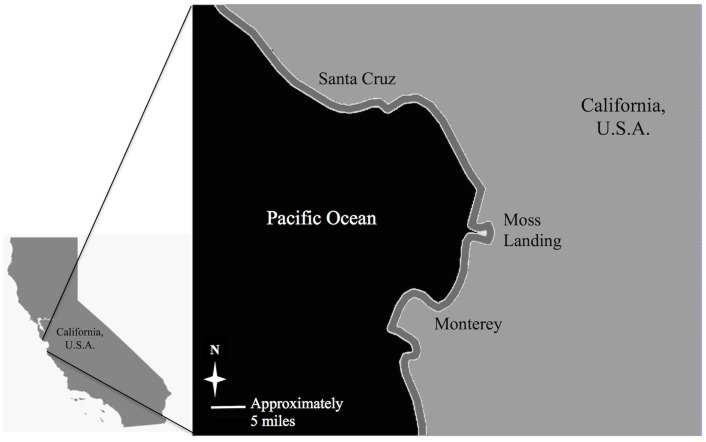
Map of the Monterey Bay area. Map includes the three major ports: Santa Cruz, Moss Landing, and Monterey.

We used annual time series data for the period 1974–2012 to assess trends in the Monterey Bay CPS finfish and squid fisheries. Our data series begins two years prior to the passage of the MSA to help contextualize the period that followed. We used landings (weight) to characterize the status of the fishery and determine fishery dominance, defined as a given species accounting for the plurality of landings in any given year. These data were extracted from the California Department of Fish and Wildlife (CDFW) commercial fisheries information system (CFIS), Table 18PUB, Poundage and Value of Monterey Bay Area Commercial Fishing, and summed across the region’s three major ports: Monterey, Moss Landing, and Santa Cruz (Fig. 2).

### Determining dominance transition points

To identify modes of dominance, or time periods when a given species accounted for the plurality of landings, we performed a cluster analysis of the log of annual landings for all three species together and identified groups based on the Bray-Curtis Similarity index, choosing 40

percent similarity as a cut off point (Primer-E v. 6, United Kingdom). To better view which years were within any one dominance mode, we created multidimensional scaling (MDS) ordination plots to map the years based on similarity in a 2-dimensional space, where years closer to one another on the plot are more similar in terms of pounds landed. We used the Many Eyes visualization software (IBM 2007) to create bubble-plots showing proportional changes in landings during identified transition points from one dominant fishery mode to another. We then used a Simpson’s diversity index comprised of landings to identify years of relatively equal catches and years focused primarily on a single species. To support the identification of dominance modes, we consulted with nine Monterey Bay wetfish fishery experts, from academia to NGOs and industry representatives [37]. With their guidance, an extensive literature review provided additional insight into characterizing the transitions between dominance modes.

### Variables associated with long-term fishery trends

We applied model fitting with forward variable selection using a set of variables to examine the extent to which landings for each species are associated with various drivers. Here we refer to the large-scale sectors that influence the fisheries, such as markets and climate as ‘drivers’, and the metrics by which to measure or identify the drivers as ‘variables’, such as local market price and Pacific Decadal Oscillation (PDO) indices. To guide the identification of possible drivers of variability and change in the fisheries, we used the four subsystems defined in Ostrom’s social-ecological system framework for common pool resources [27]. This general framework uses a common set of subsystems and variables to illuminate the complexities and external factors within any common pool resource system. Drivers we expected to be associated with long-term trends in landings include: climate dynamics (in Ostrom’s resource units subsystem), governance phase changes (in the governance subsystem), market forces (in the resource system subsystem), and participation trends (in the users subsystem). Although potentially important, we did not include technology (in the resource system subsystem) and community identity (in the resource user subsystem) drivers in the models due to lack of time series data at our geographic scale (see S2 Text for examples of how each subsystem has influenced fisheries).

We selected variables that relate to each of these drivers based on data availability and significance to this case study. We list and describe the variables used in the next section. We explored potential carry-over effects of these variables from year to year using lagged data (with one-, two-, and three-year lags) for each variable when a sufficient number of years of data was available. For each fishery, we used factor analysis including all possible independent variables and excluding the landings of the fishery in question to generate a set of factors used to test for explanatory relationships with landings of the target species. Many of these potential explanatory variables were highly correlated (e.g., ENSO and PDO, the number of fishing vessels and fishing trips), preventing detailed conclusions about the role of individual predictors. Therefore our approach (factor analysis with orthogonal axis rotation; VARIMAX, SAS 9.4 software) generated a smaller set of non-correlated factors that retain the predictive relationships of the original variables. We identified explanatory variables with significant loadings (P<0.05) on each of these factors in our analysis. To determine which of these variables were associated with factors that could explain significant portions of the variance in the landings for each fishery, we entered all factors with eigenvalues greater than one, along with categorical variables to code for the governance phases of all three species fisheries, into an ANCOVA model using forward variable selection. We retained those factors entering the model with a P-value less than 0.15 in our final model (a commonly used criteria; e.g. [38,39]) and calculated effect sizes (semi-partial Eta-squared) to determine the portion of variance accounted for by each selected factor. Model results produced an assembly of factors, representing combinations of independent variables (e.g., market price) that are significantly correlated with the dependent variable, landings.

### Details on independent variables used in model selection

**Resource Unit Subsystem Variables.** Several oceanographic variables known to affect the biology of the three study species included annual mean values for the El Niño Southern Oscillation (ENSO) and PDO indices, and seasonal averages of the Bakun upwelling index [40–43]. We calculated PDO values as the annual average of the standardized values for the PDO index, derived as the leading principal component of monthly sea surface temperature anomalies in the North Pacific Ocean, poleward of 20° North, from the University of Washington Joint Institute for the Study of the Atmosphere and Ocean (JISAO). We calculated ENSO values as the annual average Multivariate ENSO Index (MEI), obtained from the NOAA Earth Systems Research Laboratory. We included upwelling trends as the seasonal averages of the Bakun index of the 36°N 122°W observations (NOAA Pacific Fisheries Environmental Laboratory).

We used sardine biomass [44] as an indicator of that species’ ecological status. We were not able to incorporate quantitative data for the other species, as anchovy and squid biomass data were not available. However, we included qualitative observations from the literature of anchovy and squid abundance to explain trends in those fisheries.

**Governance Subsystem Variables.** We identified governance phase changes through an extensive literature search and use of expert knowledge. We focused on restricted access measures, which we believe play a vital role in effort shifts among fisheries, and divided each fishery into distinct phases marked by particular formal and informal governance events and circumstances (Table 2).

**Table 2:** Governance Phases.

Fishery	Phases	Description
**Squid**		
A	1976–1993	Fishery managed by the California Legislature
B	1994–1997	Discussion and efforts to restrict access
C	1998–2004	California SB 364 passed and implemented, with moratorium on entry and other measures California Fish and Game Commission (CFGC) assumes management of the fishery (2001)
D	2005–2012	State implements Market Squid FMP, with permanent restricted access and other measures
**Anchovy**		
A	1976–1977	CFGC manages fishery based on a reduction quota
B	1978–1999	Pacific Fishery Management Council (PFMC) adopts and implements Northern Anchovy FMP (NAFMP)
C	2000–2012	PFMC amends NAFMP, adds other CPS species to establish the CPS FMP
**Sardine**		
A	1976–1985	CDFW manages fishery, with moratorium on directed fishery, incidental catch allowed
B	1986–1990	State re-opens directed fishery with 1,000-ton annual quota
C	1991–1999	State increases quota as stocks recover Quota geographically divided between two regions Discussion of limited entry and inclusion in a federal FMP Fishery declared rebuilt
D	2000–2012	Sardine (and other wetfish species) added to CPS FMP, with limited entry south of Point Arena

**Resource Subsystem Variables.** We calculated market price from the same tables and at the same scale as landings (CDFW Table 18PUB) and present the data in US dollars, adjusted for inflation (using the 2014 consumer price index inflation calculator from the US Department of Labor). To estimate Monterey Bay area exports of each species and enable assessment of trends therein, we used landings at the three Monterey Bay area ports combined as a proportion of statewide landings, and multiplied the result by the amount exported from California. We extracted export data from the NOAA National Marine Fisheries Service database of foreign trade annual data, compiled by specific US customs districts since 1975.

**Resource User Subsystem Variables.** We evaluated trends in the number of trips and number of vessels as indicators of fishery activity for Monterey and Santa Cruz counties. We extracted these data from the Pacific Fisheries Information Network (PacFIN) database (from data originating from CDFW’s CFIS), retrieved in August 2013, from the Pacific States Marine Fisheries Commission, Portland, Oregon (www.psmfc.org). Using the CDFW landings data and PacFIN number of trips data, we also used catch per unit effort as a variable, calculated as average landings per trip for each fishery.

## Results

### Identifying and characterizing dominance transition modes

From the cluster analysis, we identified four dominant fisheries modes (in order of frequency): a) squid dominated years, b) sardine dominated years, c) anchovy dominated years, and d) low landings years for all wetfish fisheries. The first group, squid-dominated years, encompassed most years of our study period (1978–1982, 1985–1994, 2002–2003, 2010–2012). The second, sardine-dominated years, consists of two 5- to 6- year periods (1995–2001 and 2004–2009). The anchovy fishery dominated from 1975 to 1977 only. All three wetfish fisheries exhibited low landings in 1983 and 1984. The MDS plots confirmed the cluster analysis with distance indicating dissimilarity among four main phases. We identified seven years as transition points among these four phases where fishery dominance changed: 1978, 1983, 1985, 1995, 2002, 2004, and 2010.

**Fig 3:**
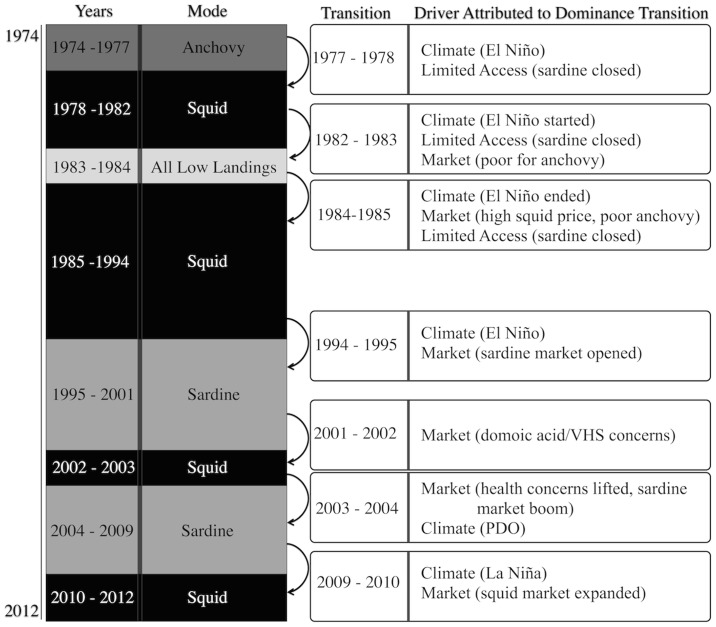
Timeline of dominance modes and transition points. Identified drivers most associated with each transition are listed accordingly.

An extensive literature review guided by the cluster analysis results indicated that over all, climate and market played the biggest role in determining dominance of a given fishery, (i.e., when one fishery accounted for a plurality of landings), or when effort shifted from one dominant fishery to another. Of the seven points of dominance transitions, shifts were most attributed to: climate (1978, 1983, 1985, 1995, 2004, 2010), market factors (1983, 1985, 1995, 2002, 2004, 2010), and limited access regulations (1978, 1983, 1985) (Fig. 3).

**Fig 4:**
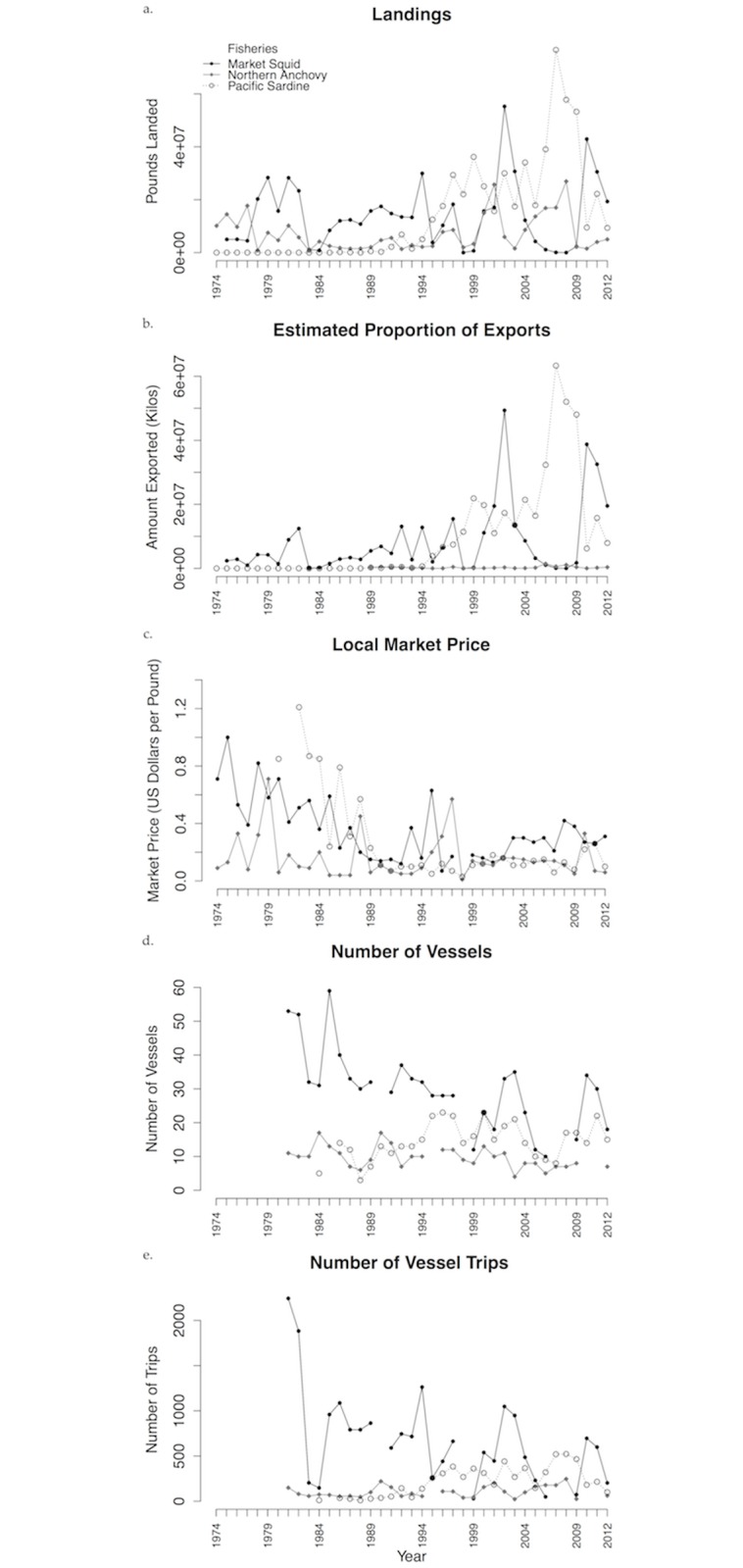
Selected variables in time series plots. The a. landings, b. estimated proportion of exports, and c. market price are values for the Monterey, Santa Cruz, and Moss Landing ports combined for the period 1974–2012, while the d. number of vessels and e. number of trips are specific to all Monterey and Santa Cruz county sites combined from 1981–2012.

The time series plots of the landings, estimated proportion of landings exported, local market price, number of vessels fishing, and number of trips (Fig. 4a–4e) provide further evidence of differing fishery dominance phases, highlighting the dominance of the market squid fishery throughout the time series. In recent years, when the squid fishery did not dominate, the sardine fishery did. Anchovy played a dominant role in landings at the very beginning of the time series, but dropped to the least dominant fishery in 1978 after demand for squid increased and the sardine fishery re-opened.

**Fig 5:**
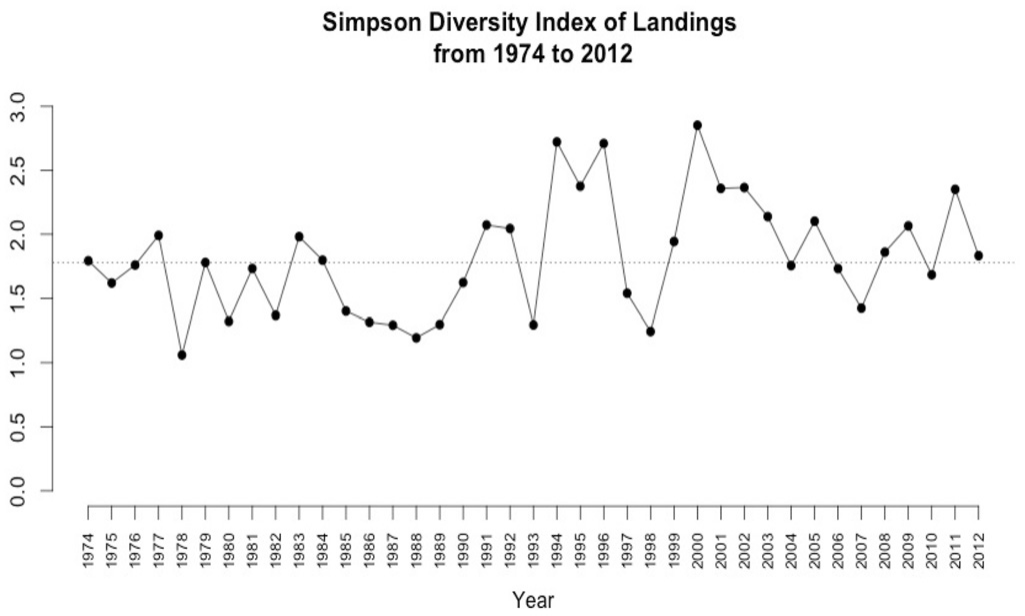
Simpson Diversity Index of landings. Higher values indicate more evenness (less dominance) among fisheries landings, lower values indicate less evenness (greater dominance of a single fishery), based on pounds landed (CDFW Table 18PUB) at the three study ports combined. Dotted line represents average.

**Fig 6:**
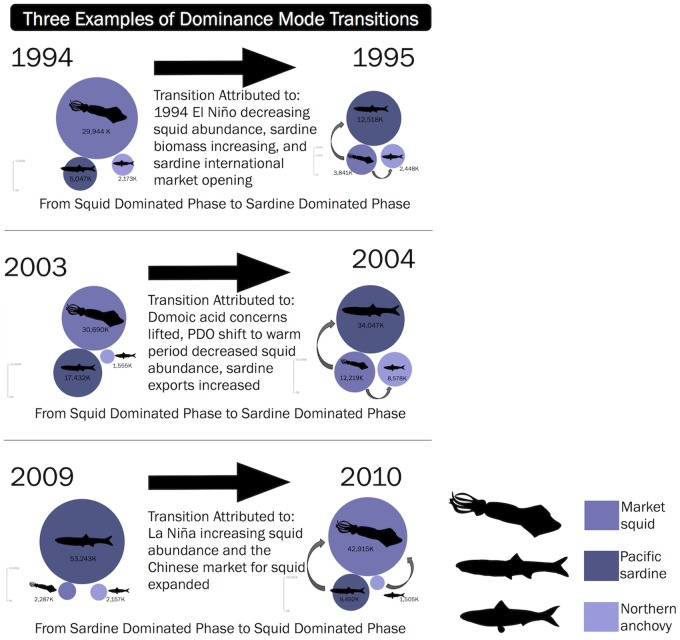
Dominance mode transition points. Proportional landings bubble plots showing dominance mode transition points for three of the seven transition years identified by the cluster analysis. Circle size is scaled to relative volume of landings (Data from Table18PUB CDFW). Darkest purple circles are sardine landings, lightest purple are anchovy landings. Gray arrows represent the movement of focus shifting from one fishery to the next.

The Simpson Diversity Index, calculated for landings, identified a dominant species in 1978, 1980, 1982, 1985–1989, 1993, 1998, and 2007 (Fig. 5). The difference among landings for these fisheries is highest in these years, with one fishery dominating the system. In 1978, 1980, 1982, 1985–1989, and 1993, squid dominated the landings, and then in 1998 and 2007, the sardine fishery yielded the highest catches. Squid dominated the system after El Niño events and during La Niña events, and sardine landings were greatest during high export years. Generally, the species perceived to be most abundant corresponded to the dominant fishery. The Simpson index also identified the three years of relative evenness—1994, 1996, and 2000—during which fishery participants clearly depended on all three fisheries combined rather than any single fishery in particular. The transition points from 1994 to 1995, 2003 to 2004, and 2009 to 2010 best visually display the shift from one fishery dominance mode to another (Fig. 6).

### Model selection: drivers for long-term fishery trends

Model fitting with forward variable selection resulted in optimized models for each species (Table 3). Changes in sardine landings were most explained by sardine governance phases and squid landings were most explained by squid governance phases. Sardine landings and sardine biomass, followed by sardine exports best explained anchovy landings. As management decisions are driven by evidence of environmental conditions and estimated abundance [33,45], the governance phases appear to be a response to perceived changes in abundance. These abundance-guided governance phases explained long-term fishing trends more than any other variables. Other important variables that explained long-term fishing trends include fishing effort metrics (i.e., CPUE and number of anchovy vessels) and climatic drivers of availability.

**Table 3:** ANCOVA Results.

Fishery	Explanatory Variable	Variation Accounted For
**Squid** R^2^ = 0.842 p-value = 0.0003	Squid Governance Phases	0.8048
	Factor 1: spring upwelling, no. of anchovy vessels	0.0375
**Anchovy** R^2^ = 0.959 p-value = 0.0002	Factor 1: sardine landings, sardine biomass, sardine exports, anchovy CPUE, sardine CPUE, fall upwelling lagged 0, 1, 2 years	0.5416
	Factor 2: no. of anchovy vessels	0.1939
	Squid Governance Phases	0.1560
	Factor 3: negative correlation with summer upwelling lagged 0 and 1 year and squid landings	0.0399
	Sardine Governance Phases	0.0275
**Sardine** R^2^ = 0.854 p-value = 0.0002	Sardine Governance Phases	0.4467
	Factor 1: anchovy landings, fall upwelling, sardine biomass, sardine CPUE, anchovy CPUE	0.3248
	Factor 2: anchovy landings lagged 3 years	0.0420
	Factor 3: squid landings lagged 1 and 2 years	0.0389
	Factor 4: negative correlations with no. of anchovy vessels and spring upwelling	0.0016

Factors are listed in order of highest contribution to explaining landings to lowest contribution to explaining landings. All significant variables are included in this table.

## Discussion

Traditional fisheries, especially small-scale fisheries, have relied on interconnected systems to maintain market and community stability, and such systems can enhance the overall sustainability and resilience of fisheries. This case study of Monterey Bay wetfish serves as an example of a modern interconnected system, and is an illustration of how flexibility supports social and ecological aspects of fisheries. Fisheries are subject to a number of possible drivers that can and do affect fishery trends. The ability to adapt to the resulting variability and change enables fishermen and buyers to support themselves and the local fishery system, particularly in economically marginal years that otherwise may lead to spatial displacement or drive participants to seek other livelihoods. Adaptation also allows for the pressure on one fishery to be reduced when conditions make it vulnerable. Other fisheries that might benefit from such an interconnected system can adopt a number of strategies to encourage the ease of shifting effort among fisheries. In fisheries that are strongly driven by factors such as climate and markets, governance systems may enable or hinder adaptability (i.e., the ability to be flexible and shift focus among fisheries). Thus, to enable the flexibility to adapt, fishery managers should understand the social-ecological dynamics and actively acknowledge possible external drivers as inherent aspects of the system as opposed to external shocks or ‘surprises’ and encourage flexibility. Promoting access through less expensive permits or less stringent regulation to multiple fisheries of an interconnected system, stimulating investment in diversifying gear types, and establishing multi-species processing at local facilities can all lead to stronger interconnected and adaptive fishing systems. While the Monterey Bay case may be a fortuitous outcome of unplanned responses by various actors, we highlight aspects that may be codified and applied elsewhere. Below we explore the details and plausible explanations for the dominance mode transitions and model selection results, followed by an overview of understanding the vitality of the Monterey Bay wetfish SSF and more general lessons on the applicability of interconnected flexibility to other fisheries.

### Contextualizing fishery modes and dominance transition points

In this case study, anchovy dominated the system from 1975 to 1977, or only when the sardine fishery was closed, the squid fishery was relatively underdeveloped, and squid landings were significantly lower than average. When the sardine fishery collapsed in 1952, fishermen turned to anchovy because they had the necessary vessels, expertise, and infrastructure to support activity in the fishery. The end of this anchovy dominated mode is most attributed to fishermen shifting their focus to increasingly abundant and higher priced squid (Fig. 3). Of the three fisheries, market squid sells for the highest price and thus it is assumed that fishermen and buyers would generally prefer to target squid when they are available in marketable quantity. Squid dominated from 1978 until 1982 and then dropped significantly in 1983 and 1984 in the wake of the 1982–1983 El Niño event. Because these species have a short life cycle (~ 9 months), their population abundance and distribution depends mainly upon climate variation [40,46].

From 1983 to 1984, all fisheries had significantly lower landings, attributed primarily to the 1982–1983 El Niño event, which resulted in low abundance for all wetfish fisheries (Fig. 3). Large-scale warming occurred along the North American west coast in January 1983 and had major ecological impacts, especially on the squid and anchovy populations [47,48]. The 1983–1984 period is an example when the interconnected social-ecological system was at high risk, despite the flexibility of fishermen to move among wetfish species. Fortunately for Monterey Bay wetfish fishermen, some were able to participate in the San Francisco Bay herring and Alaska salmon fisheries [49]. Sardine populations were rebounding and increasingly were observed and caught more in 1983 and 1984 than at any other time since the moratorium began. Although the regulations were flexible in allowing incidental catch, they did not allow the fishermen to target this resource, and therefore sardine catch could not support the wetfish fisheries system.

With a poor anchovy market, and strictly limited quotas for sardines since the fishery was re-opened in 1986, squid again dominated from 1986 until the 1994 El Niño (Fig. 3). For the first time since the 1974 moratorium, the 1995 sardine quota, dictated by biomass, was large enough that the sardine fishery remained open through the end of the season and sardine dominated the system from 1995 until 2001 [44]. The international market for California sardine fully opened in 1995 [50], and the amount and value of exports increased nearly tenfold relative to the previous decade (Fig. 4b). The sardine dominance mode ended in 2002 due to health advisories and concerns related to domoic acid toxins and viral hemorrhagic septicemia (VHS), which resulted in temporary prohibitions on the sale of sardine and anchovy for human and animal consumption, and temporary bans on exporting California sardines [51,52].

Squid dominated the system for the period 2002–2003. However, this phase of squid fishery dominance ended in 2004 as health concerns about sardines subsided and biomass as well as the market for sardine grew (Fig. 3). Additionally, the market squid fishery suffered from warmer waters attributed to a PDO fluctuation, prompting fishermen to once again target sardines [53]. Strong markets likely contributed to sardine’s dominance during this time. However, the expansion of the Chinese market demand for squid in 2009 helped mark the end of this sardine dominant mode [54]. In 2010, a strong La Niña stimulated strong squid resource conditions, and fishermen who participated in the sardine fishery in 2009 shifted their attention to the squid fishery. During all squid-dominated years, sardine biomass was relatively low [44].

### Contextualization of model selection results

While dominance mode transitions are linked to conditions at the time, model selection indicated which variables played the most significant roles for long-term trends in landings of each fishery. The squid governance phases, which were the most significant in explaining squid landings, are largely products of fluctuations in squid resource availability, as sardine governance phases are products of sardine resource availability. The state and federal management systems monitor resource availability and determine strategies based on perceived abundance, such that landings were primarily dependent on availability. Thus, identified governance phases in each fishery are tightly linked to perceived or estimated abundance and best viewed as a response to changes therein. Years with both governance phase changes and fishery dominance transitions (1978, 1985, 2004) were all associated with significant shifts in abundance of one species or another.

Anchovies had the lowest market price per pound, and thus were generally the least desirable to catch. Participation in this fishery was thus affected by trends in the squid and sardine fisheries. The squid and sardine governance phases were the most significant when abundance of these two species was high; anchovies were targeted less since fishermen could access higher-value products. These species respond to climatic changes and upwelling intensity, and the significance of the upwelling variables is an indicator of how the fisheries are environmentally driven.

### Flexibility in the Monterey Bay interconnected system

In the sardine, anchovy, and squid fisheries of Monterey Bay, we have three species fished largely by the same fleet. Fishermen and buyers generally have a choice of which fishery to participate in, and we assume that this choice largely depends on availability of the product (which depends on climate conditions), and markets (domestic and international). Most fishermen can readily change nets, and can use the same equipment, vessels, personnel, and receiving and processing infrastructure for all three species.

This multi-species network is highly variable from year to year, suggesting a need for flexible management. The interconnected system has persisted because of a number of factors including ease of gear-switching, possibility of buying operations with or qualifying for permits in all fisheries, spatial similarities, and temporal complementarity in species availability. Fishery dominance in this system changes as a result of climate, market, and limited access regulations, as well as from the interaction among these factors.

The Monterey Bay wetfish fisheries, like the majority of the world’s fisheries, do not exist in a vacuum, but are influenced by external fisheries and external users. The stocks are not restricted to the bay’s waters, and neither are the fishermen. Thus the issue of scale is critical to consider when analyzing these fisheries as well as when comparing attributes and outcomes of management strategies according to differently sized fisheries. For example, the Monterey Bay squid fleet and squid processors include actors from Monterey Bay, Port Hueneme and Ventura, and San Pedro Terminal Island [55]. In addition, Oregon and Washington fishermen, stocks, and management plans all influence Monterey Bay fisheries. This expanded view of the Monterey Bay wetfish fisheries in a broader context further reveals the complexities of a modern US fishery, as a multitude of drivers can lead fishermen to shift effort from one target species or one geographic area to a variety of other options, and highlights the need to consider the flexibility to adapt as a key element to fishery resilience and sustainability. As is the case for many fisheries, the development of this system was gradual and a result of key events and conditions. In the mid 20^th^ century, sardines were the primary target species and were the foundation of one of the largest industrial fishing systems in the world. Today’s wetfish fisheries were built on the ruins of this large industry, and its collapse allowed for this interconnected set of fisheries to emerge. With a closed sardine fishery, and before the demand for market squid grew, the focus shifted to anchovy, with fishermen and processors readily adapting fishing and receiving/processing equipment, infrastructure, and practices from the sardine fishery. Later, fishermen and buyers opportunistically responded to the opening of the Chinese market for squid, which became a major driver of fishery participation and increased emphasis on squid. Climate changes and larger market factors have altered the dynamics of the fisheries since, changing conditions and further prompting focus to shift among fisheries. Today, all three fisheries are active and the proportion of participation in each changes as environmental and market conditions vary (Fig. 6).

The Monterey Bay case study serves as a data-rich example in an industrialized country. However, many SSFs are data-poor and are in less industrialized regions. Additionally, many SSFs have different goals and structures than the Monterey Bay commercial fisheries, as many SSFs are directed for subsistence or local trade only. However, the role of adaptability in the persistence of Monterey Bay fisheries is not contingent on their commercialization or location in an industrialized country. Drivers of variability in conditions and responses are very relevant in other settings, and enabling the flexibility to adapt is still a key element for fisheries to survive such fluctuations in conditions. We were able to use the considerable amount of available information for this region to provide a concrete example of the role of flexibility in enabling adaptive capacity and contributing to the persistence of a fishery system over several decades. This case study provides some ideas for managing multi-species SSFs in diverse contexts and also presents types of data that can be collected in other systems, in order to analyze the role and trends of adaptability in those systems. While Monterey Bay is relatively data-rich, a number of drivers and other information were not available, especially at our temporal and spatial scale (see S3 Text for a more complete review of data limitations). Future collection and assimilation of such information could provide further insight into the dynamics of shifting effort among fisheries and how social-ecological multi-species SSFs adapt to fluctuating conditions.

### The role of governance in interconnected systems

Participants in other SSFs have emphasized the need for more flexibility, and this study serves as an example of how to approach analyzing the opportunities for adaptation in a fishery system [56]. We show that market and climate factors more frequently drive the shifting of focus among fisheries than governance factors. However, governance can be a key factor in limiting or enabling shifting effort among fisheries. This is especially true in the first three years of the time series, as restrictions on directed sardine fishing hampered participation in this fishery and thus the least desirable species, anchovy, dominated. This again occurred during the low landings years of 1983–1984 when prohibitions on targeting sardine kept fishermen from participating in the fishery and El Niño conditions made squid and anchovy scarce, threatening the system.

Governance is a critical factor in affecting the flexibility to adapt in the fishery and in preventing the depletion of stocks, as occurred previously in the sardine fishery. The 1950s sardine collapse destroyed the industry that depended on it [57,58]. However, through time, careful management, and a number of instrumental decisions made early on, such as implementing spatial and temporal closures, the ecosystem rebounded and the sardine fishery and many others now support the community [32,59]. Since then, the federal government, the State of California, and the fishing community have managed the fisheries in an effort to prevent another collapse. Continued monitoring and assessment of environmental conditions, the resource, and its use are the foundations for maintaining a functioning system. Thus, formal governance is now structured to respond to and enable adaptation to drivers. These drivers primarily consist of climate and market factors that can change the status and attractiveness of a fishery from one year to the next. Governance still plays an important role as new regulatory actions can lead fishermen and buyers to seek economic opportunities elsewhere, but with governance allowing for the flexibility to adapt, now climate dynamics and market variability drive the shifting of focus among fisheries.

### Management implications

It is difficult to manage fisheries adaptively for fluctuating drivers such as climate and markets, except to maintain the ability of fishermen to shift effort among three strongly interacting fisheries. There are key management implications to consider in managing each species individually versus as a portfolio. This case study strongly suggests that optimizing the management of a single species may create lock-ins, or path dependencies, that constrain the flexibility to adapt and have unintended consequences to other fisheries that are strongly related via portfolio effects [60]. Additionally, future management decisions should avoid impairing the flexibility that has led to participant’s adaptability during fluctuating conditions. Such possible decisions include closed access policies such as TURFs (Territorial Use Rights for Fishing) that can impair access to multiple fisheries through spatial limitations, especially fisheries such as these where fishing grounds rapidly shift according to changing temperatures.

The movement of fishery participants among various fisheries is key to maintaining adaptability in these fisheries. Such flexibility provides a buffer against any one fishery becoming economically or ecologically unviable. This is a major concern, especially considering the recent scholarship on the effects of individual rights-based approaches such as catch shares. The trend in US fisheries toward such approaches can reduce diversification and result in consolidation and overcapitalization, which hinders the capacity of fishery participants to shift to other alternatives [13,17]. An example of such a consolidation policy effect is seen in the British Columbia halibut fishery, where the efficiency and financial viability goals of the individual transferable quota (ITQ) system meant to benefit the fishery and community were not met and therefore distressed a number of actors in the system [61]. Other similar situations include cases where consolidation has threatened fishermen’s safety [62], innovation and debate within the fishery [63], community equity [64], generational recruitment [65], and conservation efforts [63,64]. Managing for flexibility can be used to avoid unintended consequences of fisheries management. Multi-species dedicated access systems such as multi-species catch shares have been proposed to mitigate such concerns [66,67]. Such strategies seek to minimize spatial displacement, where if people cannot shift focus to other local fisheries, they may leave the area and enter fisheries further afield. This, in turn, can drain economic resources and livelihoods away from a community and increase pressure on resources and associated human systems elsewhere [68]. Limiting fishermen to a single fishery can make fishermen vulnerable, especially if that fishery becomes economically unviable.

The best method by which to manage fisheries for flexibility is debatable. An adaptable permitting system may be needed to integrate the management of strongly interacting fisheries. This applies to many fisheries globally, where fishermen shift focus seasonally and/or annually, or due to market or climate drivers. Managing for flexibility can be framed as managing portfolios of strongly interacting small-scale fisheries, and recognizing factors that may affect such portfolios. The CPS and squid permit systems in the Monterey Bay case provide the opportunity for fishermen to access all permits needed to catch each species, thus helping to ensure economic stability for participants. Additionally, management that takes into account the conditions for success within the entire social-ecological system is benefited by shared knowledge between fishermen, scientists, and managers. In the case of the Juan Fernández lobster fishery, management practices restricted the relatively sustainable system in part because scientists and managers outside the local community were not aware of its informal tenure system [69]. This push for knowledge sharing and trust between different types of stakeholders is valuable especially for small-scale fisheries and has been a characteristic for improved fishery outcomes in some cases, such as in Chilean coastal fisheries [70,71]. Such participatory management strategies can further enhance implementing flexibility in a system as linkages and relationships may be more readily identified. One method that could further strengthen interconnected fisheries is expanding the lowest priced catch of a multi-species fishery to higher priced markets. In the case presented here, expanding the anchovy market to higher priced products would create more equal weight in desirability of the three species and may lead to more evenly distributed effort and more economic stability for participants.

The literature suggests that such systems may perform best if the interconnected system is at the same trophic level [16,72]. In some cases, the exploitation of a broad range of species results in “opportunistic depletion” where high-value, low abundance species are collected since the low-value, high-abundance species are in the same area and caught with the same gear [73]. One area of caution is when interconnected fisheries are too similar and occupy the same niche, or are susceptible to the same risks. For example, the reason this interconnected system did not collapse during the 2002 domoic acid event was because fishermen could shift effort from the sardine fishery to the squid fishery. Thus, creating an interconnected system of species that occupy different niches or differ in their biological response to risks, such as vulnerability to toxins, may provide additional resilience. Interconnected systems would be beneficial in cases where coastal development and associated higher costs of living have led to economically nonviable single species fisheries. This has been seen in cases such as the Florida Keys where management failed to consider the broader socioeconomic context when creating a high cost of entry for the traditional fisheries, resulting in the majority of spiny lobster, shrimp, and stone crab fishery participants exiting the industry as they could no longer make a living targeting one species [74]. Other types of interconnected fisheries that depend on flexibility include nearshore tropical artisanal fisheries where participants integrate mangrove, reef, and semi-pelagic fisheries into a complex multi-species, multi-ecosystem network [75]. The sectoralization of these fisheries or ecosystems could be harmful to participant's livelihoods and also may lead to ineffective management efforts. Another method for identifying potential interconnected fisheries for flexible management is in seasonal variation. In the case of the Piracicaba River fishery, the migrations of river fish are reflected in the usage of particular gear types according to seasonal patterns [76]. The flexibility to shift gears with seasonal migrations is key to maintaining a steady fish production throughout the year for these fishermen. Additional flexibility may in some cases be the recognition of fishery participants shifting efforts between the fishery and other industrial sectors [1,77–79]. The acknowledgment of such seasonal participation is critical to both fishery management and conservation initiatives, as well as for these participants who are often vulnerable and dependent on flexibility for their livelihoods.

Fishermen have historically fished the mean, knowing there will be big years and there will be lean years [24,80]. Considering all the drivers that can induce fluctuations in one or more fisheries, we believe the key to management is to encourage flexible interconnected fishery systems. Managing fisheries as a coupled social-ecological multi-species system for flexibility provides extra assurance that the fisheries and participants will remain viable within and across years, even as conditions vary and change.

## Supporting Information

S1 Text Additional information on Monterey Bay wetfish fisheries and the management system. (DOCX)

S2 Text Ostrom subsystems applied. Examples of drivers influencing fisheries as categorized by Ostrom’s social-ecological subsystems.
(DOCX)

S3 Text Data Limitations. (DOCX)
