# Supplementary material for: Managing Small-Scale Commercial Fisheries for Adaptive Capacity: Insights from Dynamic Social-Ecological Drivers of Change in Monterey Bay
Source: PLoS One. 2015 Mar 19;10(3):e0118992. doi: 10.1371/journal.pone.0118992 (PMC4366077; doi:10.1371/journal.pone.0118992)
Supplement: S1 Text — (DOCX) [file pone.0118992.s001.docx]

**S1 Text.** **Additional information on Monterey Bay wetfish fisheries and the management system**

We use the term ‘fishermen’ instead of ‘fisher’ to refer to both men and women, as most fishery participants in the study area prefer the former. The Monterey Bay area, including Santa Cruz, Monterey, and Moss Landing ports, is one of three major centers of activity for the fisheries (the other two are Los Angeles/San Pedro and Ventura/Port Hueneme) [1]. Whereas fishermen and buyers tend to operate primarily within one of those centers, most also operate within a second area [1]. Present day California wetfish fisheries have strong connections to the "traditional" or historic fishery of the early to mid 1900s [1]. Most fishermen and buyers have a long personal and family history in the fishery. Relationships among them are social as well as economic and have enabled many to withstand the challenges of variable and uncertain environmental, regulatory and economic conditions. Most of the catch from these fisheries is exported.

The Pacific Fishery Management Council (PFMC) actively manages the Pacific sardine (*Sardinops sagax*, Clupeidae) and Pacific chub mackerel (*Scomber japonicas*, Scombridae) fisheries, and monitors those for northern anchovy (*Engraulis mordax*, Engraulidae), market squid (*Loligo opalescens*, Loliginidae), and jack mackerel (*Trachurus symmetricus*, Carangidae). Pacific herring (*Clupea pallasii*, Clupeidae) and jacksmelt (*Atherinopsis californiensis*, Atherinopsidae) are important Ecosystem Component Species included in this FMP and all species of West Coast krill or euphausiids (primarily *Euphausia pacifica*, Euphausiidae and *Thysanoessa spinifera*, Euphausiidae*)* are managed as prohibited harvest species [2].

The CPS limited entry plan for finfish was established in 2000. In 2003, the CPS FMP [2] then formulated a capacity goal that provided for limited entry permit transferability. In California, the management of market squid has been under the authority of the California Fish and Game Commission since SB 209 was enacted in 2001, pursuant to the goals and requirements of California’s Marine Life Management Act (MLMA) [3]. The Market Squid Fishery Management Plan (MSFMP) implemented in 2005 includes a seasonal catch limit, monitoring programs, weekend closures, gear regulations, a restricted access program with provisions for initial entry, permit types, permit fees and transferability, as well as a seabird closure.

**References**

1. Pomeroy C, Hunter M, Los Huertos M. Socio-Economic profile of the California wetfish industry. In: Pleschner DB, editor. California’s “wetfish” industry: Its importance past, present and future. Santa Barbara: California Seafood Council; 2002. pp. 46.

2. Pacific Fishery Management Council. Coastal pelagic species fishery management plan as amended through amendment 13. Portland (OR): PFMC; 2011 Sep.

3. State of California Resources Agency. Final market squid fishery management plan. Los Alamitos (CA): California Department of Fish and Game Marine Region; 2005 Mar.
