# Supplementary material for: Managing Small-Scale Commercial Fisheries for Adaptive Capacity: Insights from Dynamic Social-Ecological Drivers of Change in Monterey Bay
Source: PLoS One. 2015 Mar 19;10(3):e0118992. doi: 10.1371/journal.pone.0118992 (PMC4366077; doi:10.1371/journal.pone.0118992)
Supplement: S2 Text — Examples of drivers influencing fisheries as categorized by Ostrom’s social-ecological subsystems. (DOCX) [file pone.0118992.s002.docx]

**S2 Text.** **Examples of drivers influencing fisheries as categorized by Ostrom’s social-ecological subsystems**

*Governance Subsystem*

Regulatory measures can create situations in which fishermen choose to pursue one fishery over another [1]. For example, in New Zealand, adoption of the Quota Management System in 1986 led to fishermen shifting effort completely over to fisheries not included in the quota system, such as tuna [2]. In the case of Monterey Bay fisheries, state and federal policies play an important role in dictating how fisheries function and which fisheries appear to be more profitable. For instance, the groundfish fishery has seen a number of limitations, from gear restrictions and time and area closures to catch limits [3].

*Users Subsystem*

User agency can play a significant role in changing conditions of any common pool resource system [4]. In Monterey Bay, fishing always has been a part of the identity of the people living in the area [5]. Due in part to John Steinbeck’s *Cannery Row*, sardines and other wetfish are often associated with Monterey Bay as a place and tourism center. This link between the fisheries and the identity of Monterey Bay may contribute somewhat to the persistence of these fisheries.

*Resource System Subsystem*

The resource system is composed of the structure and processes of the fishery. Here we focus on the market and technological aspects of this subsystem. Changing and distorted markets also haven been shown to affect fishery outcomes [6-8]. For example, the strong preference for fresh seafood and decreasing local supplies in China led to a 552 percent increase in United States exports of crab to China from 2009 to 2010 [9]. In our study, opening of the Chinese market to California squid led to significant changes in squid fishery participation [3,10]. The role of technological change is evident in other fisheries such as the Atlantic bluefin tuna fishery, where storage innovations led to that fishery’s expansion [11] as well as in the case where deepwater trawl vessels are now able to aim for previously inaccessible Patagonian toothfish [12]. In Monterey Bay, improvements and adoptions of new technologies did occur during our time series, with notable advancements including the use of fish finders, GPS, satellite imagery, and communication tools, along with the power block, the wetfish pump, and, for squid, high intensity lights. These innovations increased access to resources and fishery efficiency, but were adopted gradually over time, precluding marked changes in trends or significant dominance mode shifts.

*Resource Units Subsystem*

The role of climate in these fisheries, especially large-scale phase variations such as the Pacific Decadal Oscillation (PDO) and El Niño Southern Oscillation (ENSO), is well documented as the strongest driver of production in these fisheries over the past 50 years [13-16]. The three wetfish species respond differently to climatic events, such that fishermen shift effort among those fisheries as conditions warrant. Sardine tends to dominate during warm PDO periods, and anchovy tends to dominate during cool PDO periods. Market squid is known to be negatively affected by (warm-water) El Niño events, and anchovy tends to be more negatively affected by El Niño events than sardine. The sensitivity of squid to warmer water explains the considerable variation in landings from year to year. In addition, seasonal upwelling in Monterey Bay plays a significant role in ecosystem fluctuations [17]. Although our time series begins in 1976 in association with of a governance factor (passage of the Magnuson-Stevens Act), it coincides with one of the largest climate regime shifts (cold to warm) observed in this area [18].

**References**

1. Degnbol P, McCay BJ. Unintended and perverse consequences of ignoring linkages in fisheries systems. ICES J Mar Sci. 2007;64: 793-797.

2. Yandle T, Dewees CM. Consolidation in an individual transferable quota regime: lessons from New Zealand, 1986-1999. Environ Manage. 2008;41: 915-928.

3. Pomeroy C, Dalton M. Socio-economics of the Moss Landing commercial fishing industry. Report to the Monterey County office of economic development. Santa Cruz (CA): California Seafood Council; 2003.

4. Bodin Ö, Tengö M. Disentangling intangible social–ecological systems. Glob Environ Change 2012;22: 430-439.

5. Palumbi SR, Sotka C. The death and life of Monterey Bay: A story of revival. 1st ed. Washington DC: Island Press; 2010.

6. Berkes F, Hughes TP, Steneck RS, Wilson JA, Bellwood DR, Crona B, et al. Globalization, roving bandits, and marine resources. Science. 2006;311: 1557–1558.

7. Andrew NL, Béné C, Hall SJ, Allison EH, Heck S, Ratner BD. Diagnosis and management of small-scale fisheries in developing countries. Fish Fish. 2007;8: 227-240.

8. Anderson SC, Flemming JM, Watson R, Lotze HK. Rapid global expansion of invertebrate fisheries: trends, drivers, and ecosystem effects. PLoS One. 2011;6: e14735.

9. Bean R, Han A. Live crab and lobster exports boom. USDA Foreign Agricultural Service Global Agricultural Information Network (GAIN) Report. Beijing: USDA; 2011 Jun.

10. Pomeroy C, Hunter M, Los Huertos M. Socio-Economic profile of the California wetfish industry. In: Pleschner DB, editor. California’s “wetfish” industry: Its importance past, present and future. Santa Barbara: California Seafood Council; 2002. pp. 46.

11. Sumaila UR, Huang L. Managing bluefin tuna in the Mediterranean Sea. Mar Policy. 2012;36: 502-511.

12. Ainley DG, Brooks C, Eastman J, Massaro M. Unnatural selection of Antarctic toothfish in the Ross Sea, Antarctica. In: Huettmann F, editor. Protection of the three poles. Tokyo: Springer; 2012. pp. 53-73.

13. Jacobson L, MacCall A. Stock-recruitment models for Pacific sardine (Sardinops sagax). Can J Fish Aquat Sci. 1995;52: 566–577.

14. Dalton MG. El Niño, expectations, and fishing effort in Monterey Bay, California. J Environ Econ Manage. 2001;42: 336-359.

15. Chavez FP, Ryan J, Lluch-Cota SE, Niquen CM. From anchovies to sardines and back: multidecadal change in the Pacific Ocean. Science. 2003;299: 217-221.

16. Koslow JA, Allen C. The influence of the ocean environment on the abundance of market squid, doryteuthis (loligo) opalescens, paralarvae in the Southern California Bight. Cal Coop Ocean Fish. 2011;52: 205-213.

17. Breaker LC, Broenkow WW. The circulation of Monterey Bay and related processes. Oceanogr Mar Biol. 1994;32: 1-64.

18. Overland JE, Alheit J, Bakun A, Hurrell JW, Mackas DL, Miller AJ. Climate controls on marine ecosystems and fish populations. J Mar Syst. 2010;79: 305-315.
