# Supplementary material for: Managing Small-Scale Commercial Fisheries for Adaptive Capacity: Insights from Dynamic Social-Ecological Drivers of Change in Monterey Bay
Source: PLoS One. 2015 Mar 19;10(3):e0118992. doi: 10.1371/journal.pone.0118992 (PMC4366077; doi:10.1371/journal.pone.0118992)
Supplement: S3 Text — (DOCX) [file pone.0118992.s003.docx]

**S3 Text.** **Data** **Limitations**

While some social factors are not readily quantifiable [1], this study was limited, as many are, in incorporating information from the users and community perspective over a 38-year time frame. The lack of time series data on the social aspects of Monterey Bay fisheries limits this type of analysis. We attempted to collect information on the number of fishermen, the portfolio of all local fishermen over time (fishery plurality), employment outside the fishing industry (occupational plurality), and the location of catches, but data consistent with the temporal span and spatial scale of this study are not readily available. Time series data on squid and anchovy biomass and recruitment, the Gini index of income for the larger community over time, annual income per fisherman, annual fishermen demographics, and membership information for fishery organizations (to further understanding of community involvement) were unavailable for our entire time period at our three ports/two counties. Such data would enable assessment of the role of the community as a driver, would provide finer resolution of the fishery system, and would afford a more complete understanding of fishermen’s income diversity. Previous studies have generated data and insights to inform our approach and facilitate interpretation of our results [2,3]. Additionally, data for the smaller Monterey Bay ports could not be included as such information is not publically available due to small numbers and thus confidentiality rules. However, the three ports included in our study account for nearly all wetfish fishing activity over the study period.

**References**

1. Plaganyi ÉE, van Putten I, Hutton T, Deng RA, Dennis D, Pascoe S, et al. Integrating indigenous livelihood and lifestyle objectives in managing a natural resource**.** Proc Natl Acad Sci U S A. 2013;110: 3639-3644.

2. Pomeroy C, Dalton M. Socio-economics of the Moss Landing commercial fishing industry. Report to the Monterey County office of economic development. Santa Cruz (CA): California Seafood Council; 2003.

3. Pomeroy C, Hunter M, Los Huertos M. Socio-Economic profile of the California wetfish industry. In: Pleschner DB, editor. California’s “wetfish” industry: Its importance past, present and future. Santa Barbara: California Seafood Council; 2002. pp. 46.
